# Mitogen-Inducible Gene 6 Inhibits Angiogenesis by Binding to SHC1 and Suppressing Its Phosphorylation

**DOI:** 10.3389/fcell.2021.634242

**Published:** 2021-02-22

**Authors:** Lixian Liu, Liying Xing, Rongyuan Chen, Jianing Zhang, Yuye Huang, Lijuan Huang, Bingbing Xie, Xiangrong Ren, Shasha Wang, Haiqing Kuang, Xianchai Lin, Anil Kumar, Jong Kyong Kim, Chunsik Lee, Xuri Li

**Affiliations:** State Key Laboratory of Ophthalmology, Zhongshan Ophthalmic Center, Sun Yat-sen University, Guangzhou, China

**Keywords:** MIG6, angiogenesis, SHC1, endothelial cell, ocular neovascularization

## Abstract

The mitogen-inducible gene 6 (MIG6) is an adaptor protein widely expressed in vascular endothelial cells. However, it remains unknown thus far whether it plays a role in angiogenesis. Here, using comprehensive *in vitro* and *in vivo* model systems, we unveil a potent anti-angiogenic effect of MIG6 in retinal development and neovascularization and the underlying molecular and cellular mechanisms. Loss of function assays using genetic deletion of *Mig6* or siRNA knockdown increased angiogenesis *in vivo* and *in vitro*, while MIG6 overexpression suppressed pathological angiogenesis. Moreover, we identified the cellular target of MIG6 by revealing its direct inhibitory effect on vascular endothelial cells (ECs). Mechanistically, we found that the anti-angiogenic effect of MIG6 is fulfilled by binding to SHC1 and inhibiting its phosphorylation. Indeed, SHC1 knockdown markedly diminished the effect of MIG6 on ECs. Thus, our findings show that MIG6 is a potent endogenous inhibitor of angiogenesis that may have therapeutic value in anti-angiogenic therapy.

## Introduction

The blood vessel network is vital for both normal physiology and numerous diseases. Blood vessels not only transport oxygen and nutrient to tissues and cells required for their functions and maintenance, they also have unique functions by serving as a regulator of vascular tone, organ development, immunity, and blood-organ communication (Karaman et al., 2018; Li and Carmeliet, 2018). However, uncontrolled growth of new blood vessel can result in life-threatening pathologies, such as cancer and many other neovascular diseases (Apte et al., 2019; Li et al., 2019). Angiogenesis therefore needs to be tightly controlled to avoid overproduction of blood vessels. Currently, the majority of the studies have focused on angiogenic factors. Much knowledge in this aspect has been gained and anti-angiogenic therapies targeting angiogenic factors, such as VEGF, have been used to treat patients with neovascular diseases (Apte et al., 2019). However, despite the great success, drug resistance can develop over time, suggesting the involvement of multiple factors and pathways, such as genetic instability of tumor cells that up-regulates pro-angiogenic factors to overcome the inhibitor (Ribatti, 2016; Haibe et al., 2020). Given the presence of diverse and abundant angiogenic factors, endogenous anti-angiogenic factors would be constantly required to keep the angiogenic factors in check but less is known in this aspect. Indeed, decreased or the lack of the expression of anti-angiogenic factors may often be the reason of pathological neovascularization (Murugeswari et al., 2008; Zhang et al., 2020). Studying such endogenous anti-angiogenic molecules is therefore of critical importance.

MIG6 is a ubiquitously expressed cytoplasmic adaptor protein that modulates many cell surface receptors (Zhang and Vande Woude, 2007; Sasaki et al., 2018). MIG6 is involved in diverse biological events, including suppressing the epidermal growth factor receptor (EGFR) pathway (Ferby et al., 2006; Zhang and Vande Woude, 2007; Park et al., 2015). However, MIG6 has many EGFR-independent functions. For example, MIG6 induces cell cycle arrest in an EGFR-independent manner (Sasaki et al., 2018). MIG6 is widely expressed in various types of cells and tissues (Jin et al., 2007; Zhang and Vande Woude, 2007; Anastasi et al., 2016), including the vascular system (Jin et al., 2009; Lee et al., 2014). The expression of MIG6 is rapidly induced by various growth factors and cellular stresses under pathological conditions (Zhang and Vande Woude, 2007). However, it remains thus far unknown whether it plays a role in angiogenesis.

Here, we utilized both knockout mice and cultured endothelial cells and examined the effect of MIG6 on angiogenesis. We found that MIG6 is a potent endogenous inhibitor of angiogenesis. Genetic deletion of *Mig6* increased retinal angiogenesis in mice, and its overexpression inhibited hypoxia-induced retinal neovascularization. Overexpression of MIG6 reduced aortic and choroidal microvessel growth, and suppressed endothelial cell proliferation, migration and sprouting. Mechanistically, we show that the anti-angiogenic effect of MIG6 is exerted by inhibiting SHC1 signaling. Our thus findings reveal a new function for MIG6 as an endogenous inhibitor of angiogenesis, which may have implications in anti-angiogenic therapy.

## Materials and Methods

### Mice

All animal experiments were approved by the Animal Use and Care Committee of Zhongshan Ophthalmic Center, Sun Yat-sen University (2015-098). The *Mig6* knockout mice were kindly provided by Dr. George Vande Woude at Van Andel Institute (Grand Rapids, MI, USA). *Mig6* knockout mice used for experiments were bred on C57Bl/6J background for more than six generations and littermates were used for experiments.

### Cell Culture and Reagents

The primary human retinal endothelial cells (HREC) were purchased from Angio-Proteomie (Boston, MA, USA) and cultured in endothelial cell medium (ScienCell Research, Carlsbad, CA, USA) containing endothelial cell growth supplement (ECGS), 5% FCS, and penicillin/streptomycin. HREC within 8 passages were used for experiments.

### Retinopathy of Prematurity (ROP) Mouse Model

C57Bl6J mice at post-natal day 7 (P7) were exposed to 75% oxygen for 5 days, after which the mice were intravitreally injected with 1 μl of adenovirus expressing GFP (Ad-GFP, CV10001, 1.0 × 10^13^ pfu/ml, Vigene Biosciences, Rockville, MD, USA) or adenovirus expressing human MIG6 (Ad-MIG6, VH894726, 1.0 × 10^13^ pfu/ml, Vigene Biosciences, Rockville, MD, USA), and then returned to normoxia for additional 5 days. Mouse eyes were collected at P17 and the retinal neovascularization areas were analyzed using whole mount retinae stained with IB4-Alexa 488 (I21411, Invitrogen, Waltham, MA, USA). Neovascularization areas were outlined and quantified as the percentage of the total area of the retina using ImageJ (NIH, Bethesda, MD, USA).

### Isolation and Culture of Murine Primary Vascular Endothelial Cells

Murine primary endothelial cells were isolated from lungs of 6-week old mice. Lungs were harvested and digested with collagenase type I (17100017, ThermoFisher Scientific, Waltham, MA, USA). The digested tissues were pelleted and resuspended in PBS with 0.1% BSA and incubated with rat anti-mouse CD31 conjugated Dynabeads at room temperature for 15 min. The bead-bound cells were recovered using a magnetic separator and resuspended in complete culture medium (DMEM containing 20% FBS, supplemented with 100 μg/ml heparin, 100 μg/ml ECGS, non-essential amino acids, L-glutamine and antibiotics). Primary ECs within 3 passages were used for experiments.

### siRNA Knockdown and Adenovirus Infection of Endothelial Cells

For siRNA knockdown, ECs were transfected with human MIG6 siRNA (5′-CUACACUUUCUGAUUUCAA-3′) (Liu et al., 2012), human SHC1 siRNA (5′ CUACUUGGUUCGGUACAUGGG-3′) (Lundgren et al., 2006), or non-targeting scrambled negative control (Ribobio, Guangzhou, China) using ESCORT III (L3037, Sigma). For adenoviral infection, ECs were infected with Ad-MIG6 or Ad-GFP at an MOI of 10 for 48 h.

### Antibodies

Antibodies used in Western blots were as the following: anti-β-actin (A5316, Sigma), anti-tubulin (T6734, Sigma), anti-MIG6 (WH0054206M1, Sigma), anti-pTyr239/240 SHC1 (2434, Cell Signaling), anti-SHC1 (610878, BD Bioscience), anti-phospho-p44/42 MAPK (9101, Cell Signaling), anti-p44/42 MAPK (9102, Cell Signaling), anti-GST (2625, Cell Signaling), and anti-HA tag (A01244, GenScript). Immunoreactivity was detected using horseradish-peroxidase (HRP)-conjugated secondary antibody (RAG0072 for anti-rabbit, GAM0072 for anti-mouse, 1:5,000 dilution, Multi Sciences, Hangzhou, China).

### Construction of SHC1 Deletion Mutants

The cDNA encoding human SHC1 in pcDNA 3.1 (+) vector was obtained from GenScript (Piscataway, NJ, USA) and subcloned into a pLV-3xHA vector (Inovogen, Chongqing, China). The deletion mutants of SHC1 ΔPTB (deletion of amino acids 30–210 corresponding to protein tyrosine binding domain), ΔPro-rich (deletion of amino acids 300–366 corresponding to proline-rich domain), and ΔSH2 (deletion of amino acids 377–469 corresponding to SH2 domain) were generated using a Quickchange site-directed mutagenesis kit (Agilent Technologies) according to the manufacturer's instructions. The sequences of the oligonucleotides used to generate Shc1 mutants are as follows:

(1) ΔPTB
  CCGGACTCAGATCTCGAATT (forward)
  TACCCGGTAGAATTATCTAGGGATC (reverse)
(2) ΔPro-rich
  ATCCAGAAGTCCGCAAACAGTCGGTGTCCATGG CTGAG (forward)
  CTGTTTGCGGACTTCTGGA (reverse)
(3) ΔSH2
  CTGAGCAGCTCCGAGGGGAGGAGCGGAAACTGTC TAGAGG (forward)
  CTCCCCTCGGAGCTG (reverse)

The deletion mutants were verified by sequencing and expressed in 293T cells by transfection using Fugene 6 (Promega, Madison, WI, USA). Two days after transfection, pull-down assays were performed to examine the binding of MIG6.

### Purification of GST Fusion Protein and Pull-Down Assays

The glutathione S-transferase (GST) fusion MIG6 protein was generated by expressing human full-length MIG6 cDNA subcloned into a pGEX-4T-1 (GE Healthcare Life Sciences) in *E. coli*. For GST-Mig6 pull-down assay, 2 μg of GST-MIG6 fusion protein was added to 30 μl of glutathione magnetic beads (L00327, GenScript) and incubated at 4°C for 2 h. The protein-bound beads were then washed three times with RIPA buffer (R0010, Solarbio, Beijing, China) and incubated with the cell lysates of 293T cells transfected with SHC1 plasmids at 4°C for overnight. The beads were then washed with RIPA buffer and subjected to Western blot analysis.

### Co-immunoprecipitation of MIG6 and SHC1

HRECs were homogenized in RIPA lysis buffer with a protease and phosphatase inhibitor tablet (88668, Thermo Fisher Scientific) following EGF (50 ng/ml, PeproTech) stimulation for 30 min. The cell lysates were incubated with an anti-MIG6 antibody (sc-46167, Santa Cruz Biotechnology) for overnight at 4°C and precipitated using immobilized protein A/G plus-agarose (sc-2003, Santa Cruz Biotechnology). Immunoprecipitated protein complexes were subjected to Western blot analysis.

### Cell Proliferation and Viability Assay

Cell proliferation was determined using a Click-iT EdU Imaging Kit with Alexa Fluor 594 (C10086, Invitrogen) according to the manufacturer's instruction. Images were obtained and analyzed by ImageJ. For cell viability, 5 × 10^3^ cells of MIG6-knockdown or MIG6-overexpressing HRECs were seeded in 96-well plates. The cells were incubated for 24 h and viable cells were assessed using an MTT (3-(4,5-dimethylthiazol-2-yl)-2,5-diphenyltetrazolium bromide, CT02, Sigma) method.

### Tube Formation Assay

96-well plates were coated with 50 μl/well of growth factor reduced Matrigel (356230, BD Biosciences). The plates were incubated at 37°C for 30 min to allow Matrigel to polymerize. The HRECs were suspended in serum-free endothelial medium after treatment with siMIG6 or siMIG6 + siSHC1 and subsequently plated at 10,000 cells/100 μl/well on top of Matrigel in triplicates. After 6 h, the formation of tube-like structures was taken and analyzed using ImageJ.

### EC Spheroid Sprouting Assay

3.2 × 10^5^ of HRECs (800 cells/spheroid) were trypsinized and suspended in EC medium (ScienCell Research) containing 20% methylcellulose (Methocel, M0512, Sigma). 100 μl/well of HRECs were in a round-bottom 96-well plate and incubated overnight for spheroid formation. EC spheroids were resuspended in collagen I solution (800 μl of collagen, 200 μl of 10x M199 media, 240 μl of 0.25N of NaOH, 200 μl 0.2M of HEPES, 360 μl of H_2_O) and added 50 μl collagen solution in a 96-well plate and incubate at 37°C for 20 min. EC spheroids were centrifuged at 300 × g and resuspended in media after collecting from a 96-well plate. 80% Methocel (80% Methocel + 20% FBS) with collagen I solution were mixed and resuspended EC spheroids and transfer to a 96-well plate. After incubation at 37°C for 15 min, serum-free media was added on top of the gel. After 24 h, the images were obtained and the sprouts and their total length were analyzed using an ImageJ.

### Aortic Ring Assay

Aorta from *Mig6* knockout mice and wild-type littermates were excised and the surrounding tissues were removed. After aortic rings were cut into pieces (1 mm in length) and were placed on the top of growth factor-reduced Matrigel (354230, Corning) in 24-well plates and incubated for 7 days. Images of individual explants were obtained using a phase-contrast microscope, and converted to binary mode using a low-pass filter and threshold transformation using ImageJ. The areas of sprouting microvessels were quantified using ImageJ.

### Choroid Sprouting Assay

Mouse eyes were enucleated from wild-type and *Mig6* knockout mice. After the cornea and the lens were removed, the choroid-scleral complex was separated from the retina and cut into ~1 × 1 mm pieces. The choroid-scleral complex was placed in growth factor-reduced Matrigel (354230, Corning) for a week. The areas of sprouting microvessels were quantified using ImageJ.

### Immunofluorescence Staining and Analysis of Mouse Retinal Vasculature

The mouse retinal vascularized area of wild-type and *Mig6* knockout mice at P5 was stained using Alexa fluor 488-conjugated IB4 (I21411, Invitrogen) and anti-Erg (ab92513, Abcam) antibody. DAPI (D3571, Thermo Fisher Scientific) was used for nuclear staining. Images were acquired and the vascularized retinal areas quantified using ImageJ.

### Transwell Cell Migration Assay

HRECs (7.5 × 10^4^) in serum-free media were seeded to the upper chamber of the transwell system with 8 μm pore size (3422, Corning) for 24 h after 2 days infection with Ad-Control or Ad-MIG6. Each 50 ng/ml of either EGF (PeproTech) or VEGFA (100-20, PeproTech) was added in the bottom chamber. After 24 h, the bottom chamber was stained with DAPI (Thermo Fisher Scientific) and images were taken to analyze. The number of migrated cells per area was quantified using ImageJ.

### Statistical Analysis

Comparisons between two groups were analyzed using an unpaired or paired Student's *t*-test (two-tailed) using GraphPad Prism (GraphPad Software, La Jolla, CA, USA). Data are presented as mean ± SEM. *p* < 0.05 was considered statistically significant.

## Results

### MIG6 Is Expressed in Various Types of Vascular Endothelial Cells (ECs) From Different Human Organs

To obtain insight into the expression of MIG6 in vascular endothelial cells, we searched the public database Human Protein Atlas (www.proteinatlas.org), in which the expression levels of MIG6 are derived from published single cell RNA sequencing studies. We found that MIG6 is expressed in ECs from various tissues/organs, including skin, liver, prostate, lung, heart muscle, eye, placenta, and testis (Supplementary Table 1), indicating a potential effect of MIG6 on ECs.

### Genetic Deletion of *Mig6* Increases Retinal Angiogenesis

**Figure 1:**
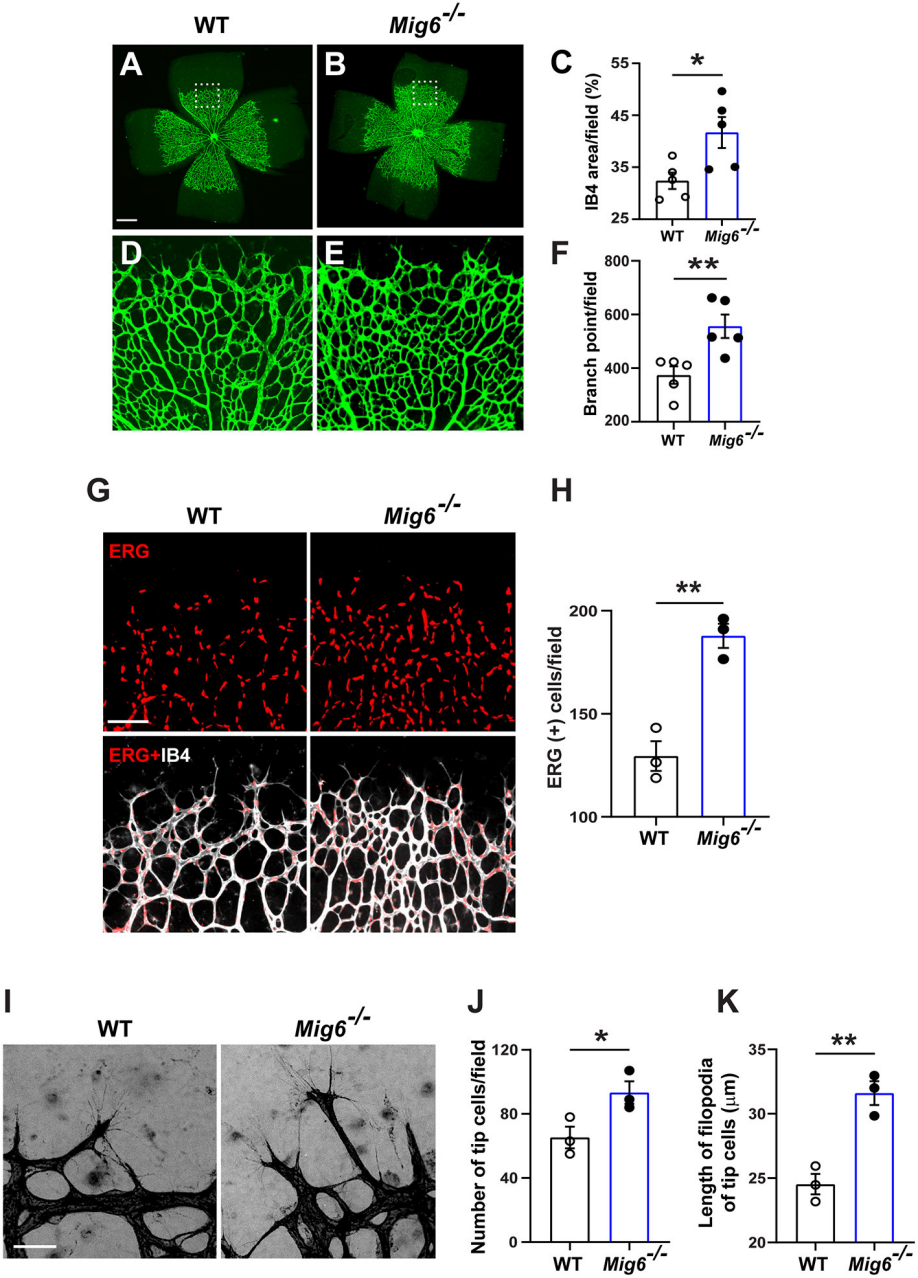
Increased retinal angiogenesis in *Mig6* deficient mice. **(A,B)** Representative images of whole mount retinae of wild-type (WT) and *Mig6*^−/−^ mice at P5 stained for IB4 (green). **(C)** Quantification of retinal blood vessel densities stained with IB4 in WT and *Mig6*^−/−^ mice (*n* = 5 fields per retina). **(D,E)** Higher magnification of dotted regions in whole mount retinae of WT **(D)** and *Mig6*^−/−^ **(E)** mice. **(F)** IB4 staining (green) in **(D)** and **(E)** showing more vascular branch points in the retinae of *Mig6*^−/−^ mice at P5 (*n* = 5 fields per retina). **(G)** Whole mount retinae co-stained for ERG (red) and IB4 (white) to label endothelial cells in WT and *Mig6*^−/−^ mice at P5. **(H)** Quantification of ERG^+^ ECs in the front of retinal vascular plexus (*n* = 3 fields per retina) in WT and *Mig6*^−/−^ mice at P5. **(I)** Images of retinal blood vessel tip cells and their filopodia extensions at the angiogenic front of WT and *Mig6*^−/−^ retinae at P5. **(J)** Quantification of the number of retinal blood vessel tip cells per field (*n* = 3 fields per retina) in WT and *Mig6*^−/−^ mice at P5. **(K)** Measurement of the length of filopodia of tip cells per field (*n* = 3 fields per retina). Scale bars: 50 μm for **(A)** and **(I)**; 100 μm for **(G)**. Data represent mean ± SEM. * *p* < 0.05, ** *p* < 0.01 (two-tailed paired Student's *t*-test).

MIG6 has also been shown to be expressed in mouse vascular system (Zhang and Vande Woude, 2007; Jin et al., 2009). However, it remains unknown thus far whether it plays a role in angiogenesis. To address this, we evaluated vascular formation in the retinae of *Mig6* knockout mice. Isolectin B4 (IB4) staining showed that *Mig6* knockout mice displayed higher retinal blood vessel density and branch points at postnatal day 5 (P5) (Figures 1A–F), while no difference was found in total retinal areas (Supplementary Figure 1A). Immunofluorescence staining of ERG, an endothelial cell (EC) nuclear marker, revealed a higher number of ECs in the retinae of *Mig6* knockout mice (Figures 1G,H). Notably, more tip cells and longer filopodia extensions of tip cells, known to be critical for angiogenesis (Gerhardt et al., 2003; Ochsenbein et al., 2016), were found in the retinae of *Mig6* knockout mice (Figures 1I–K). Together, these findings show that loss of *Mig6* increased retinal angiogenesis.

### MIG6 Inhibits Aortic and Choroidal Microvessel Growth

**Figure 2:**
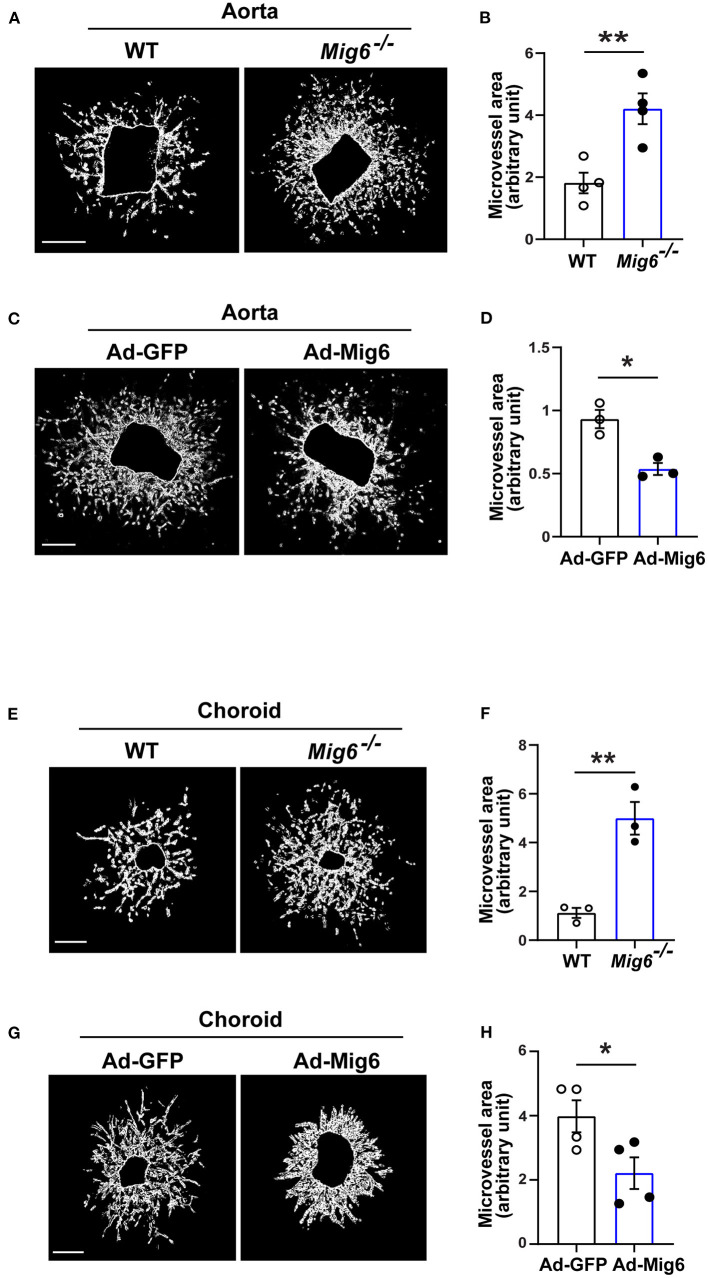
MIG6 suppresses microvessel outgrowth. **(A)** Mouse aortic rings from WT or *Mig6*^−/−^ aorta incubated for 7 days. **(B)** Quantification of vascular sprouting area per aortic ring in **(A)**. *n* = 4 aortic rings per group. **(C)** Mouse aortic rings infected with Ad-GFP or Ad-MIG6 and incubated for 7 days. **(D)** Quantification of vascular sprouting area per aortic ring in **(C)**. *n* = 3 aortic rings per group. **(E)** Mouse choroidal tissues of WT or *Mig6*^−/−^ mice incubated for 7 days. **(F)** Quantification of vascular sprouting area per choroid tissue in **(E)**. *n* = 3 choroids per group. **(G)** Mouse choroidal tissues infected with Ad-GFP or Ad-MIG6 and incubated for 7 days. **(H)** Quantification of vascular sprouting area per choroid tissue in **(G)**. *n* = 3 choroids per group. Scale bars: 500 μm for **(A)**; 250 μm for **(C,E,G)**. The data are shown as the mean ± SEM from at least three independent experiments. * *p* < 0.05, ** *p* < 0.01 (two-tailed paired Student's *t*-test).

We next investigated whether MIG6 affected angiogenesis in other tissues. An aortic ring assay showed that gene deletion of *Mig6* significantly increased microvessel growth (Figures 2A,B), while MIG6 overexpression by adenovirus markedly inhibited microvessel sprouting (Figures 2C,D). Choroidal neovascularization is a devastating pathology that can cause blindness (Shao et al., 2013). We therefore tested whether MIG6 affected choroidal angiogenesis using a choroidal sprouting assay. We found that *Mig6* deficient choroids gave rise to more microvessels than those of wild type (WT) choroids (Figures 2E,F), whereas MIG6 overexpression significantly inhibited choroidal microvessel sprouting (Figures 2G,H). Thus, apart from the retina, MIG6 also inhibits angiogenesis in other tissues, such as in the aorta and choroids.

### MIG6 Inhibits Endothelial Cell Proliferation, Migration, and Tube Formation

**Figure 3:**
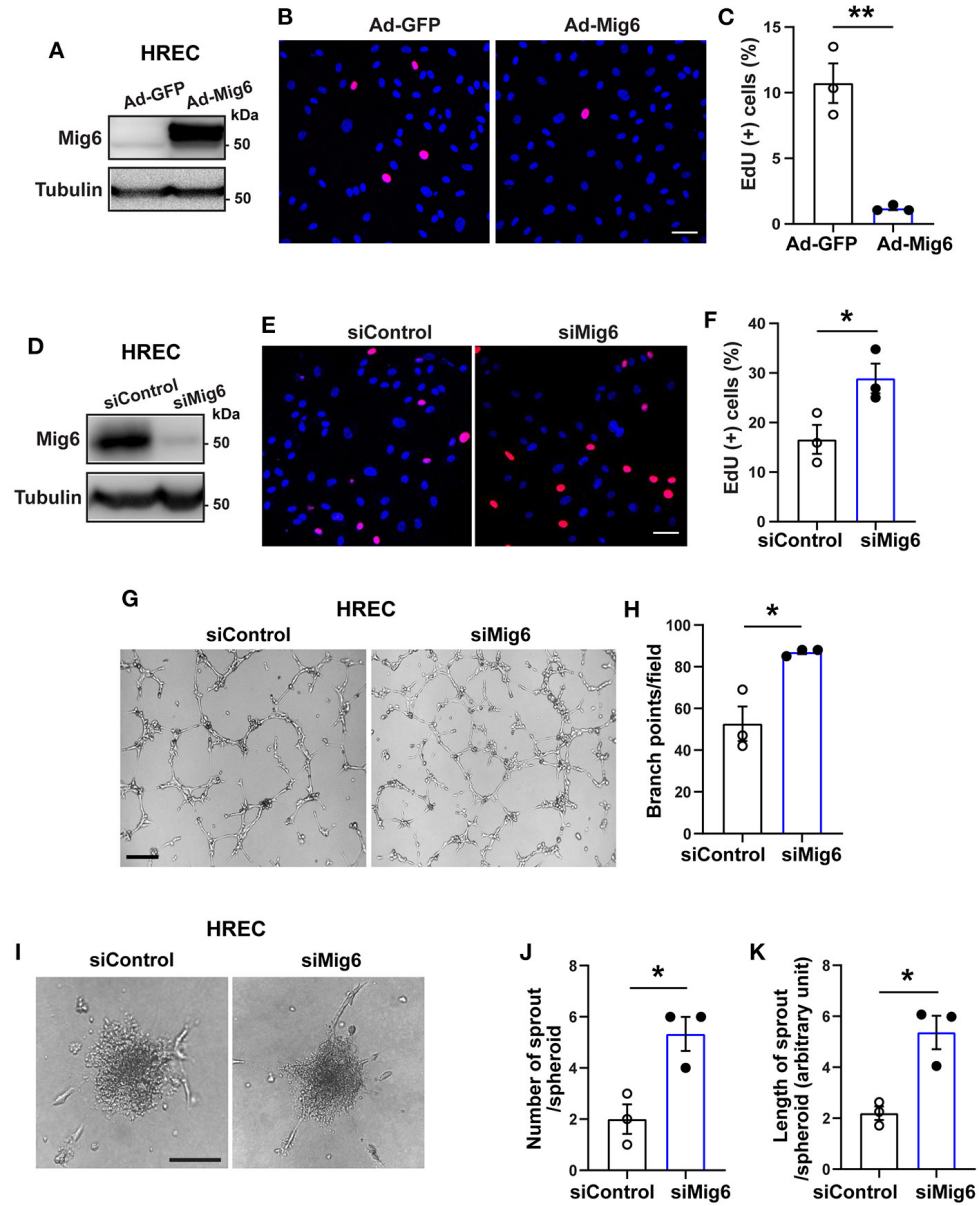
MIG6 inhibits endothelial cell proliferation, migration, tube formation, and sprouting. **(A)** Western blot for MIG6 overexpression in HRECs treated with Ad-MIG6. **(B)** Representative images for EdU incorporation in MIG6-overexpressing HRECs. **(C)** Bar graphs represent the mean ± SEM of % EdU+ cells per field in **(B)**. **(D)** Western blot for MIG6 expression in MIG6 knockdown HRECs. **(E)** Images showing EdU incorporation in MIG6 knockdown HRECs. **(F)** Bar graphs represent the mean ± SEM of % EdU+ cells per field in **(E)**. **(G)** Tube formation in HRECs upon control (siControl) or MIG6 siRNA (siMIG6)-mediated knockdown. **(H)** Quantification of the number of the branch points per field in **(G)**. **(I)** Representative images of EC spheroids using siMIG6 HREC for 48 h. **(J)** Quantification of the number of sprouts per EC spheroid in **(I)**. **(K)** Quantification of the total sprout length per EC spheroid in **(I)**. Scale bars: 50 μm for **(B,E)**; 100 μm for **(G)**; 500 μm for **(I)**. The data are shown as mean ± SEM from three independent experiments. * *p* < 0.05, ** *p* < 0.01 (two-tailed paired Student's *t*-test).

Although MIG6 is expressed in various types of endothelial cells (Supplementary Table 1) (Jin et al., 2009; Lee et al., 2014), it remains thus far unknown whether it regulates EC functions. We therefore investigated into this using both gain- and loss-of-function assays. An EdU incorporation assay revealed that Mig6 overexpression (Figure 3A) markedly inhibited the proliferation of human retinal endothelial cells (HREC) (Figures 3B,C), while MIG6 knockdown (Figure 3D) increased HREC proliferation (Figures 3E,F). This finding was further supported by an MTT assay showing that MIG6 knockdown increased and MIG6 overexpression decreased HREC proliferation, respectively (Supplementary Figures 1B,C). Moreover, an HREC migration assay showed that overexpression of MIG6 inhibited HREC migration (Supplementary Figures 2A–C), and MIG6 knockdown enhanced HREC tube formation (Figures 3G,H). Furthermore, an HREC spheroid assay revealed that MIG6 knockdown markedly increased the number and length of EC sprouts in HREC (Figures 3I–K). Thus, multiple assays showed that MIG6 overexpression inhibits HREC proliferation, migration, and tube formation.

### MIG6 Inhibits Ischemia-Induced Retinal Neovascularization

**Figure 4:**
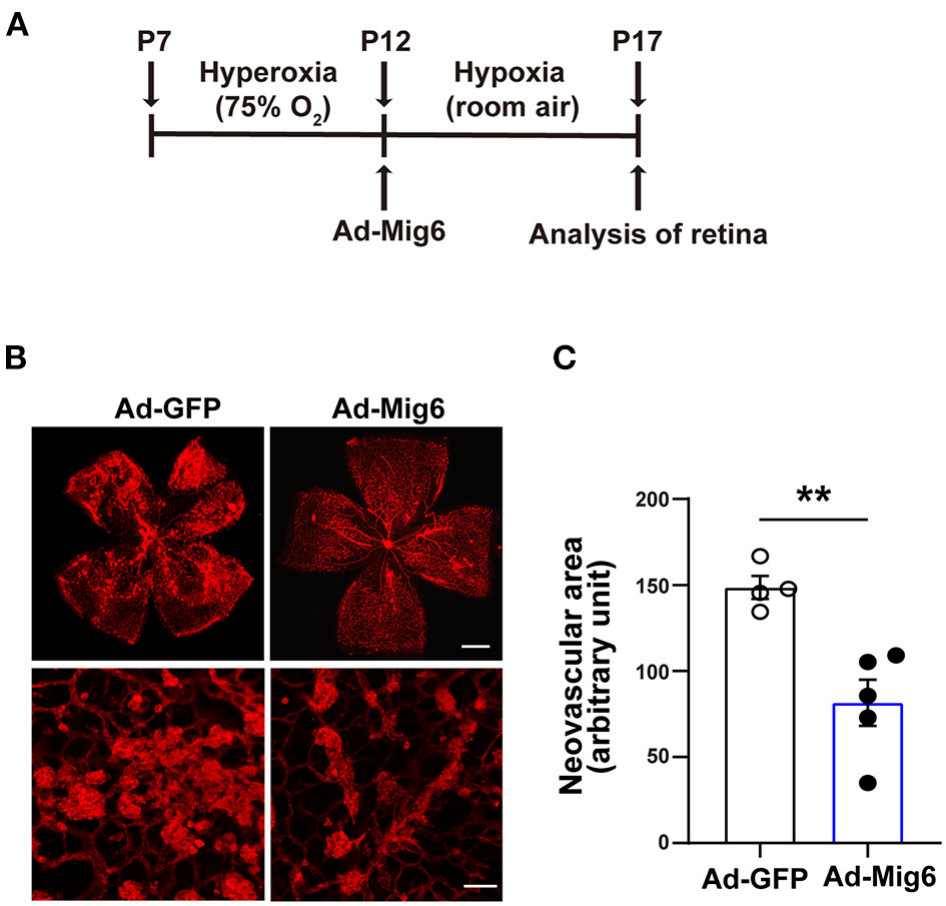
MIG6 inhibits pathological angiogenesis. **(A)** Timeline of the retinopathy of prematurity model. P7 neonatal mice were exposed to hyperoxia for 5 days. Intravitreal injection of adenovirus expressing GFP or MIG6 was executed at P12. Mice were then returned to room air until P17. **(B)** Images of whole mount retinae at P17 stained with IB4-Alexa 594 (top). High magnification images of neovascular tufts in the retinae infected with Ad-GFP or Ad-MIG6 (bottom). **(C)** Quantification of neovascular areas in retinal whole mounts in **(B)**. *n* = 4 fields per Ad-GFP treated retina, *n* = 5 fields per Ad-Mig6 treated retina. Scale bars: 300 μm for the top panel of **(B)**; 50 μm for the bottom panel of **(B)**. The data are shown as mean ± SEM. ** *p* < 0.01 (two-tailed paired Student's *t*-test).

Led by our observation on the anti-angiogenic effect of MIG6, we further tested whether MIG6 could inhibit pathological neovascularization using a mouse model of retinopathy of prematurity (ROP) (Connor et al., 2009) (Figure 4A). Adenoviruses encoding MIG6 were intravitreally injected at P12 to overexpress MIG6, with Ad-GFP as a control (Supplementary Figure 3). After 5 days, the retinae were collected to analyze neovascularization. The retinae treated with Ad-MIG6 displayed less neovascularization and fewer neovascular tufts compared with the Ad-GFP retinae (Figures 4B,C), demonstrating that MIG6 overexpression inhibits retinal neovascularization.

### MIG6 Binds to SHC1

**Figure 5:**
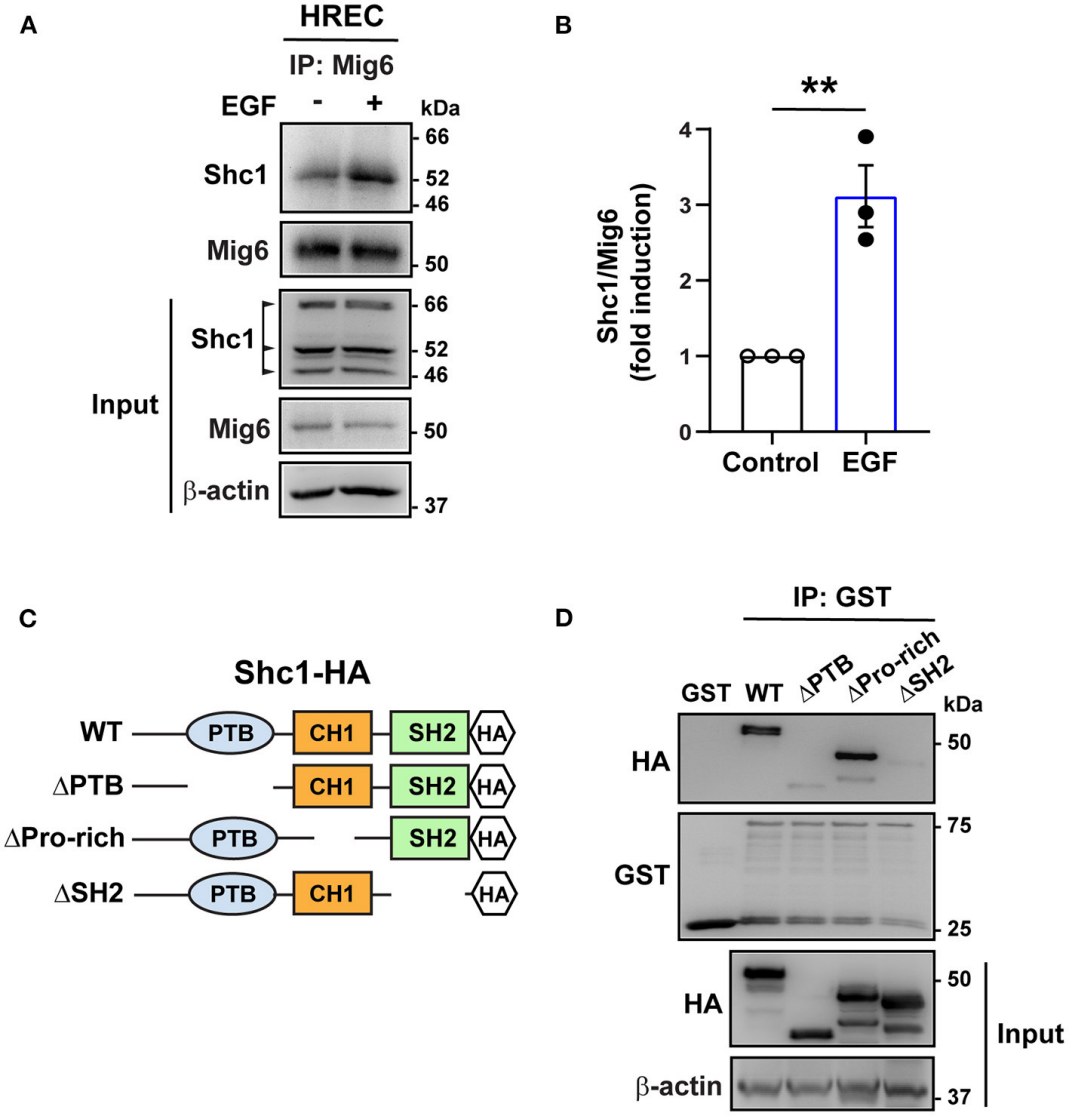
MIG6 binds to SHC1 through PTB and SH2 domains. **(A)** Immunoprecipitation (IP) followed by Western blot showing binding of MIG6 with SHC1 in HRECs, which was further augmented by EGF (50 ng/ml) treatment. **(B)** MIG6 binding to SHC1 was analyzed by densitometry and normalized by total MIG6. Fold induction relative to the control is shown as the mean ± SEM from three independent experiments. ** *p* < 0.01 (two-tailed paired Student's *t*-test). **(C)** Schematic representation of SHC1 deletion mutants. The full-length (WT) and the truncated deletion mutants of SHC1 were tagged with HA in their C-terminus. The truncated mutants of SHC1 lack the PTB domain (ΔPTB: deletion of amino acids 30–210), proline-rich domain (ΔPro-rich: deletion of amino acids 300–366) in CH1 (collagen homology 1) region, and the SH2 domain (ΔSH2: deletion of amino acids 377–469). **(D)** Association of MIG6 with the truncated mutants of SHC1 was determined by GST pull-down assay, showing that MIG6 binding is mediated by the PTB and SH2 domains in SHC1.

SHC1 has a central role in the signaling of many tyrosine kinases (Zheng et al., 2013; Ahn et al., 2017) and binds to the pY[I/E/Y/L][X][I/L/M] motif (X representing any of the 20 amino acids), which is found in MIG6 (YYLL: 394Tyr-397Leu) (Wills and Jones, 2012; Suen et al., 2013). We therefore tested whether it binds to MIG6. A co-immunoprecipitation assay revealed that SHC1 formed complex with MIG6 in HREC, which was augmented by EGF treatment (Figures 5A,B). To determine the domain of the SHC1 bound by MIG6, GST-conjugated MIG6 fusion protein (GST-MIG6) and the truncated mutants of SHC1 protein were produced (Figure 5C, Supplementary Figures 4A,B). The GST-MIG6 protein bound to the full-length and deletion mutant of SHC1 lacking the proline-rich domain (deletion of amino acids 300–366) but not to the mutants lacking PTB (deletion of amino acids 30–210) or SH2 domain (deletion of amino acids 377–469) (Figure 5D), suggesting that the PTB and SH2 domains of SHC1 are critical for MIG6 binding.

### MIG6 Inhibits SHC1 Phosphorylation

**Figure 6:**
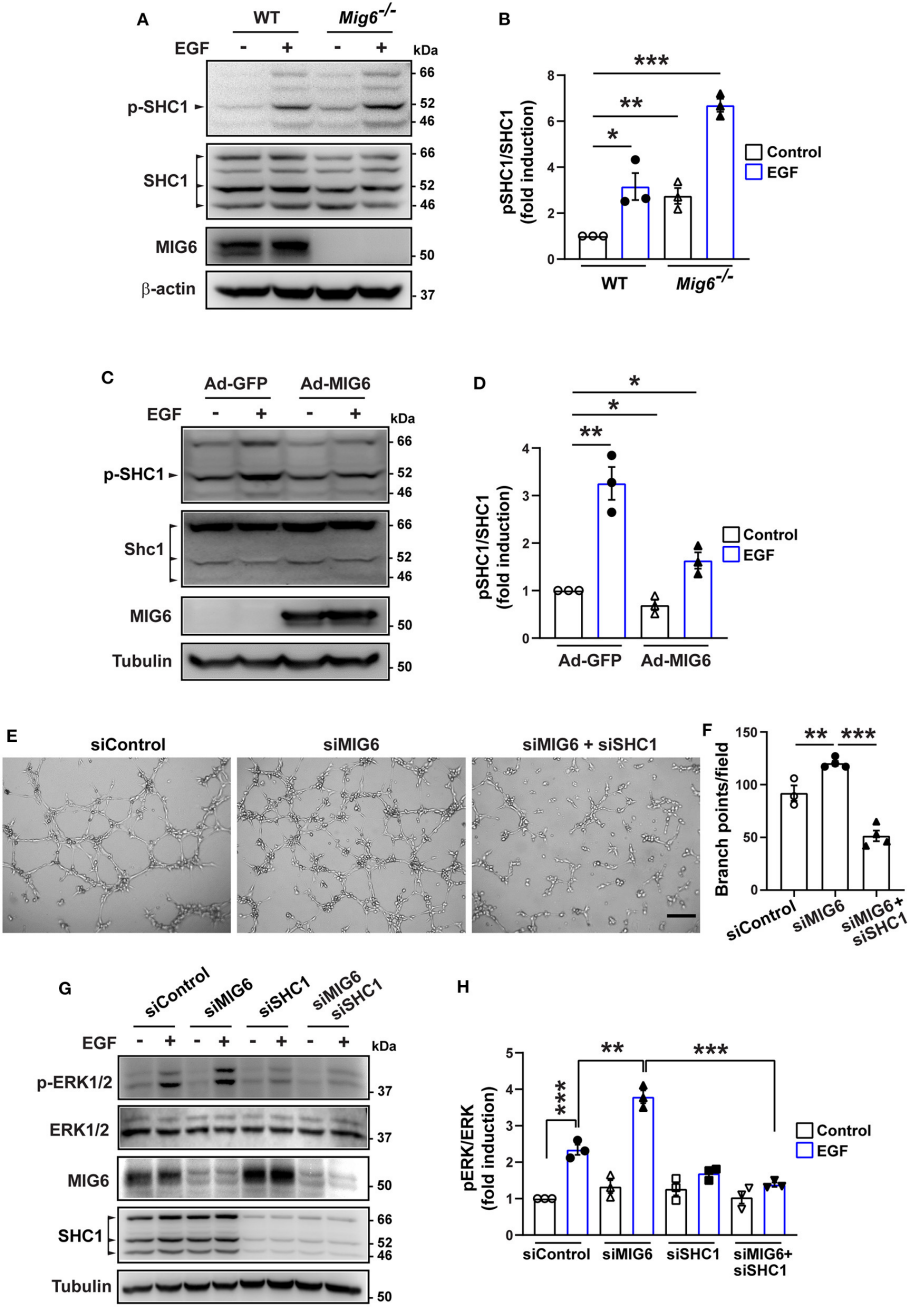
MIG6 has a potent anti-angiogenic effect by inhibiting SHC1 phosphorylation and the subsequent ERK1/2 activation. **(A)** Western blots showing that gene deletion of *Mig6* increases SHC1 phosphorylation in mouse primary lung ECs at the baseline level and in the presence of EGF. **(B)** Tyrosine phosphorylation of SHC1 (pTyr239/240 SHC1) was analyzed by densitometry and normalized by total SHC1. Fold induction relative to the control is shown as the mean ± SEM from three independent experiments. **(C)** Western blots showing that overexpression of MIG6 decreases SHC1 phosphorylation in HRECs at the baseline level and in the presence of EGF. **(D)** Tyrosine phosphorylation of SHC1 (pTyr239/240 SHC1) was analyzed by densitometry and normalized by total SHC1. Fold induction relative to the control is shown as the mean ± SEM from three independent experiments. **(E)** Images of tube formation assay showing that SHC1 knockdown abolished siMIG6-induced tube formation of HRECs. **(F)** Quantification of the branch points per field in **(E)**. The graph is shown as the mean ± SEM from three independent experiments. **(G)** Western blots showing that SHC1 knockdown abolished siMIG6-induced ERK1/2 phosphorylation in HRECs. **(H)** Phosphorylation of ERK1/2 was analyzed by densitometry and normalized by total ERK1/2. Three isoforms of SHC1 are indicated by the arrowhead **(A,C)**. Fold induction relative to the control is shown as the mean ± SEM from three independent experiments. *p < 0.05, **p < 0.01, ***p < 0.001 (two-tailed paired Student's *t*-test).

It is known that phosphorylation of SHC1 is critical in promoting cell proliferation, migration, and survival (Zheng et al., 2013; Ahn et al., 2017; Wright et al., 2019). We therefore examined whether MIG6 affected SHC1 phosphorylation. We found that gene deletion of *Mig6* increased SHC1 phosphorylation at both the basal level and after EGF stimulation in primary mouse lung ECs (Figures 6A,B). Moreover, MIG6 overexpression decreased SHC1 phosphorylation in HRECs (Figures 6C,D), while opposite effects were observed after MIG6 knockdown (Supplementary Figures 5A–D). These data thus demonstrate that MIG6 has a critical suppressive effect on SHC1 phosphorylation.

**Figure 7:**
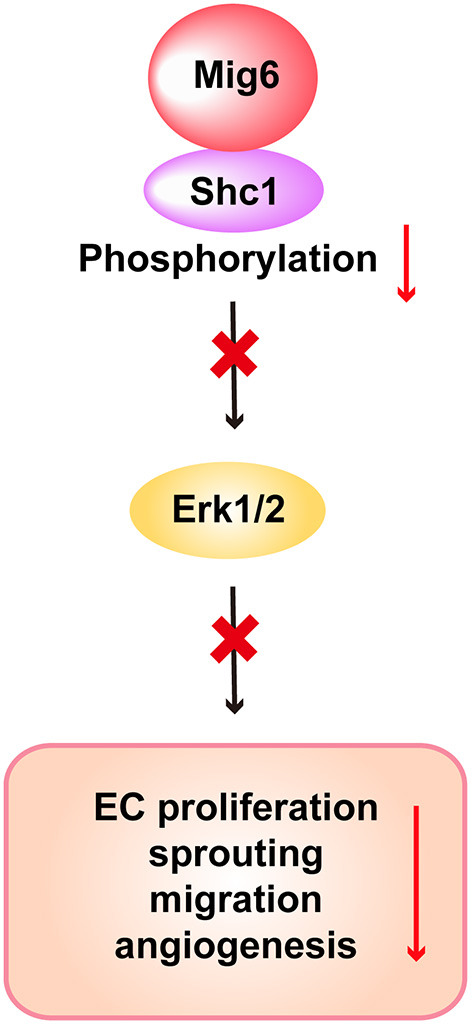
Scheme of the proposed function of MIG6 and the underlying mechanism in endothelial cells. Shown is the proposed working model displaying that MIG6 restrains SHC1 signaling pathway by binding to it and inhibiting its phosphorylation, leading to reduced ERK1/2 activation and EC proliferation and migration, thus resulting in the inhibition of angiogenesis.

We next verified whether SHC1 played a role in modulating MIG6 function. We found that SHC1 knockdown by siRNA markedly reduced MIG6 knockdown-induced tube formation in HRECs (Figures 6E,F), demonstrating that SHC1 is required for the inhibitory effect of MIG6 on angiogenesis. Moreover, at a molecular level, we found that SHC1 knockdown also abolished siMIG6-induced ERK1/2 activation in the presence of EGF in HREC (Figures 6G,H). Taken together, our data show that MIG6 has a potent anti-angiogenic effect by binding to SHC1 and inhibiting its phosphorylation (Figure 7).

## Discussion

Uncontrolled growth of blood vessels can result in many devastating neovascular diseases. Therefore, a tight regulation of angiogenesis is essential to prevent overgrowth of blood vessels and consequential exacerbation or development of neovascular diseases. Given the presence diverse and abundant angiogenic factors, naturally occurring endogenous anti-angiogenic factors would be critical to counteract excessive pro-angiogenic activities to maintain vascular homeostasis. Yet, currently, less is known about such endogenous anti-angiogenic factors as opposed to the pro-angiogenic factors. In this study, we identified MIG6 as a potent endogenous inhibitor of angiogenesis by investigating the functions of MIG6 in multiple experimental systems. Furthermore, we reveal the molecular mechanism underlying the anti-angiogenic functions of MIG6, which implicates the inhibition of SHC1 signaling driven by MIG6 binding-mediated inhibition of SHC1 phosphorylation.

MIG6 is widely expressed in vascular cells (Supplementary Table 1) (Jin et al., 2009; Lee et al., 2014). Yet, little is known whether MIG6 functions in them. Here, we found *Mig6* knockout mice displayed increased blood vessel density and number of branch points in the retinae, demonstrating an anti-angiogenic effect of MIG6 in retinal vascularization. Indeed, *in vitro*, MIG6 inhibits EC proliferation, viability and sprouting. It remains unclear thus far whether MIG6 regulates pathological neovascularization. We found in this work that overexpression of MIG6 suppressed retinal neovascularization in a mouse model of retinopathy of prematurity, providing evidence for a role of MIG6 in pathological neovascularization. It has been shown that *Mig6* deficiency led to endometrial hyperplasia (Jin et al., 2007) and neointimal hyperplasia (Lee et al., 2014), thus raising the possibility that MIG6 deficiency-induced angiogenesis might contribute to these pathological conditions. Further studies are needed to verify this.

Our findings of the anti-angiogenic effects of MIG6 present different observations from another gene knockout study, which reported the opposite roles of MIG6 in angiogenesis by showing that neovascularization is reduced compared with wild-type lungs, and pro-angiogenic factors, including VEGF-A, are downregulated at P3 in *Mig6* knockout lungs (Jin et al., 2009). At least one of the reasons for this discrepancy might be due to differential expression of MIG6 in different tissues during development (Jin et al., 2009). Additionally, in our current work, the *Mig6* knockout mice used were bred on C57Bl/6J background for more than six generations. In the Jin et al. (2009) study, however, it was not clearly indicated whether C57Bl6 strain was used. In addition, the Mig6 knockout mice used in the Jin *et al*. study were produced by crossing *Mig6*^*fl*/*fl*^ with Rosa26-Cre-ERT2, which was a different targeting strategy compared with that of our knockout mice, which is global knockout without any *Cre* recombination.

However, in line with our findings, vascular smooth muscle cells (SMCs) in SMC-specific *Mig6* conditional knockout mice displayed an increased cell migration and proliferation (Lee et al., 2014). Due to this SMC phenotype, it cannot rule out the possibility that some of the effect of MIG6 deletion on angiogenesis could be secondary to SMC defect, if any. It indeed has been reported that the EC-SMC interplay affects collective EC movements driving capillary elongation in the aortic ring assay (Arima et al., 2011). On the other hand, our HREC proliferation, tube formation and spheroid assays showed a direct effect of MIG6 on them. Also, ERG (an EC marker) staining showed more ERG+ cells in *Mig6* KO mice, indicating that MIG6 has a direct effect on ECs. Moreover, MIG6 activities vary since it interacts with a wide range of receptor tyrosine kinases (RTK), such as c-Met, FGFR2, and PDGFR (Pante et al., 2005; Zhang and Vande Woude, 2007; Borad et al., 2014; Migliore et al., 2018). Furthermore, a recent study showed that Akt is a novel binding partner of MIG6 to modulate its activation in several types of cancer cells expressing a low level of EGFR (Cairns et al., 2018). As such, by interacting with different signaling molecules depending on their expression status, MIG6 may appear to be multi-functional in different cell types or tissues.

The signaling pathway of MIG6 is poorly understood thus far. In this study, we found that MIG6 forms complex with SHC1, an intracellular adaptor protein that is highly expressed in the vascular system (Lai and Pawson, 2000; Sweet et al., 2012). Moreover, we show that the PTB and SH2 domains in SHC1 are critical regions for MIG6 binding, which leads to the inhibition of the SHC1 downstream signaling. Gene deletion of *Mig6* increased SHC1 phosphorylation in ECs, demonstrating the inhibitory effect of MIG6 on SHC1 phosphorylation. Importantly, SHC1 knockdown largely abolished MIG6 depletion-induced EC tube formation and the increased ERK1/2 activation by EGF in ECs, suggesting that the anti-angiogenic function of MIG6 is mediated by its suppressive effect on SHC1. Indeed, SHC1 has been shown to be pro-angiogenic by promoting EC proliferation, survival and blood vessel maturation (Saucier et al., 2004; Sweet et al., 2012). Noteworthy, SHC1 has a critical role in inducing VEGF expression (Saucier et al., 2004) and enhancing the activities of several angiogenic pathways, including VEGFR2 (Lai and Pawson, 2000; Sweet et al., 2012), raising the question whether MIG6 affects the angiogenic activities of the VEGFA-VEGFR2 pathway. Future studies are needed to address this.

In summary, we demonstrate that MIG6 deficiency increases angiogenesis both *in vivo* and *in vitro*. We also show that MIG6 antagonizes SHC1 signaling to inhibit angiogenesis. Our results demonstrate that the signaling axis of MIG6 and SHC1 plays a critical role in keeping angiogenesis balanced. Our work provides new insights into the pathogenesis of neovascular diseases, and may have therapeutic implications in anti-angiogenic therapy.

## Data Availability Statement

The original contributions presented in the study are included in the article/Supplementary Material, further inquiries can be directed to the corresponding authors.

## Ethics Statement

The animal study was reviewed and approved by Animal Use and Care Committee of Zhongshan Ophthalmic Center, Sun Yat-sen University.

## Author Contributions

LL and LX designed and performed experiments, analyzed data, and wrote a part of the manuscript. RC, YH, JZ, LH, BX, XR, SW, HK, and XLin performed experiments and analyzed data. AK and JK provided critical experimental tools and suggestions. CL and XLi designed and supervised experiments, analyzed data, and wrote the manuscript. All authors contributed to the article and approved the submitted version.

## Conflict of Interest

The authors declare that the research was conducted in the absence of any commercial or financial relationships that could be construed as a potential conflict of interest.
