# Supplementary material for: Mitogen-Inducible Gene 6 Inhibits Angiogenesis by Binding to SHC1 and Suppressing Its Phosphorylation
Source: Front Cell Dev Biol. 2021 Feb 22;9:634242. doi: 10.3389/fcell.2021.634242 (PMC7937727; doi:10.3389/fcell.2021.634242)
Supplement: Supplementary file 1 [file Table_1.docx]

# Supplementary Table 1.

**MIG6 is expressed in various types of vascular endothelial cells (ECs) from different human organs.**

| **EC types** | **MIG6 expression (pTPM)** |
| --- | --- |
| Skin EC | 91.1 |
| Liver EC | 70.6 |
| Prostate EC | 46.2 |
| Lung EC | 30.8 |
| Heart EC | 23.6 |
| Eye EC | 14.5 |
| Placenta EC | 8.5 |
| Testis EC | 3.1 |

From The Human Protein Atlas: <https://www.proteinatlas.org/ENSG00000116285-ERRFI1/celltype>

pTPM: protein-coding transcripts per million
