# Supplementary material for: Mitogen-Inducible Gene 6 Inhibits Angiogenesis by Binding to SHC1 and Suppressing Its Phosphorylation
Source: Front Cell Dev Biol. 2021 Feb 22;9:634242. doi: 10.3389/fcell.2021.634242 (PMC7937727; doi:10.3389/fcell.2021.634242)
Supplement: Supplementary file 2 [file Data_Sheet_1.docx]

Supplementary Material

**
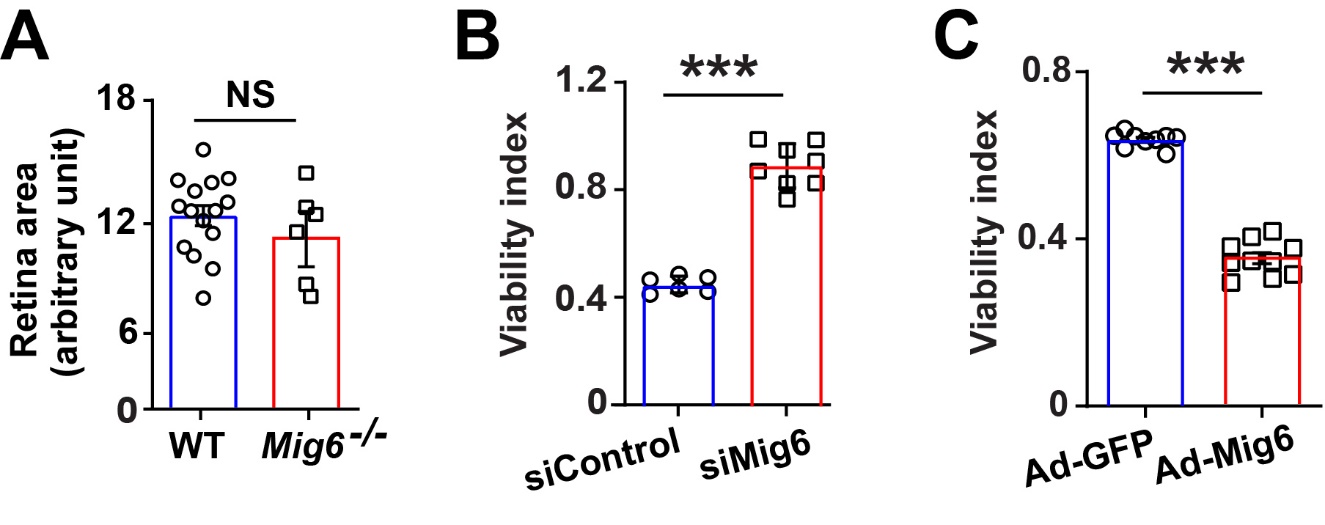
**

**Supplementary Figure 1.** Mig6 inhibits EC sprouts and vascular outgrowth. (**A**) Data are shown that there is no difference in total retinal area between wild type (WT) and Mig6^-/-^ mice. (**B**) Mig6 knockdown (siMig6) increased HREC proliferation in an MTT assay. (**C**) Mig6 overexpression (Ad-Mig6) decreased HREC proliferation in an MTT assay. The data are shown as the mean ± SEM from three independent experiments. *** *p* < 0.001, NS: not significant (paired Student’s t-test, two-tailed).


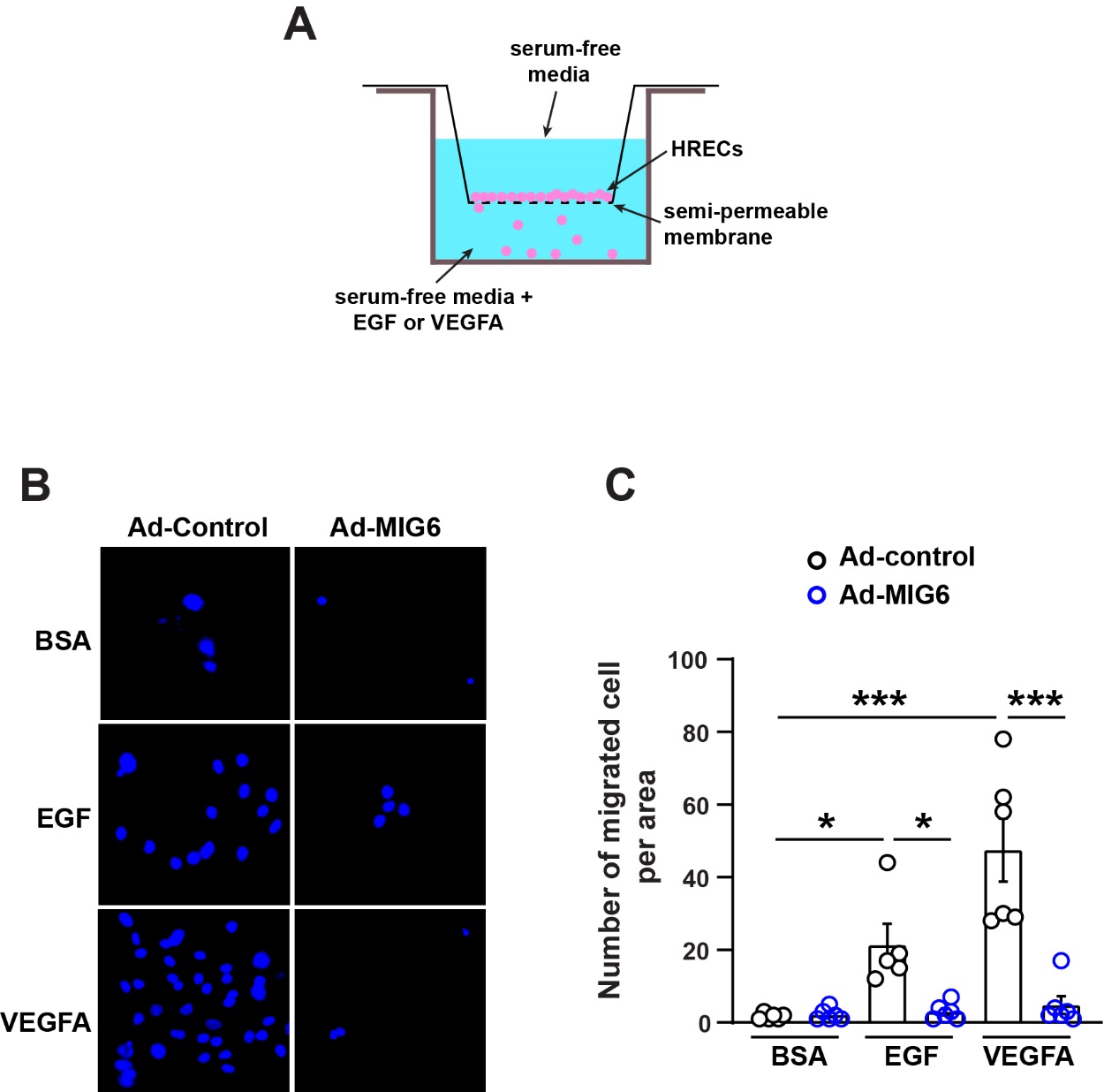


**Supplementary Figure 2.** Mig6 overexpression inhibits EGF- or VEGFA-induced HREC migration. (**A**) Schematic image of Trans-well migration assay. HREC (7.5 x 10^4^) was seeded in the top chamber after two days infection with Ad-control or Ad-MIG6. Each 50 ng/ml of either EGF or VEGFA was added into the bottom chamber. (**B**) Representative DAPI staining images of HREC migration infected with Ad-Control or Ad-Mig6. (**C**) Quantification represents the number of migrated cells infected with Ad-GFP or Ad-Mig6. The data are shown as the mean ± SEM from three independent experiments. * *p* < 0.05, *** *p* < 0.001 (paired Student’s t-test, two-tailed).

**
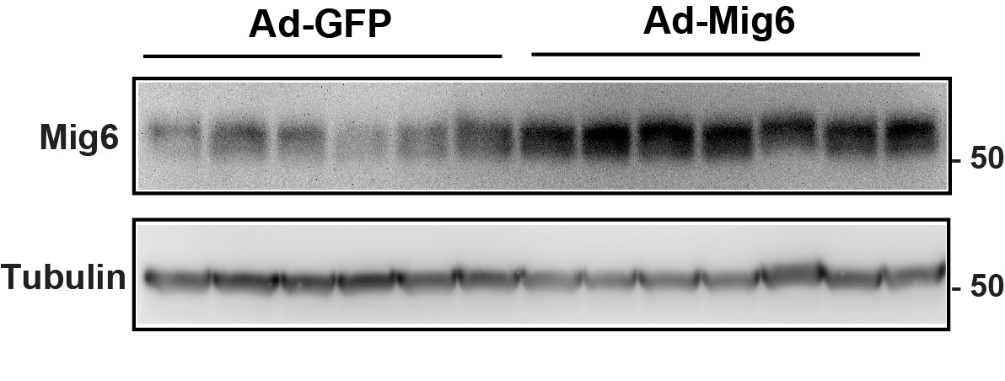
**

**Supplementary Figure 3.** Mig6 expression in mouse retina by intravitreally injected adenoviruses. Adenoviruses expressing Mig6 (Ad-Mig6) were intravitreally injected into the mouse eyes at P12 and incubated for 5 days. Ad-GFP was used as a control.

**
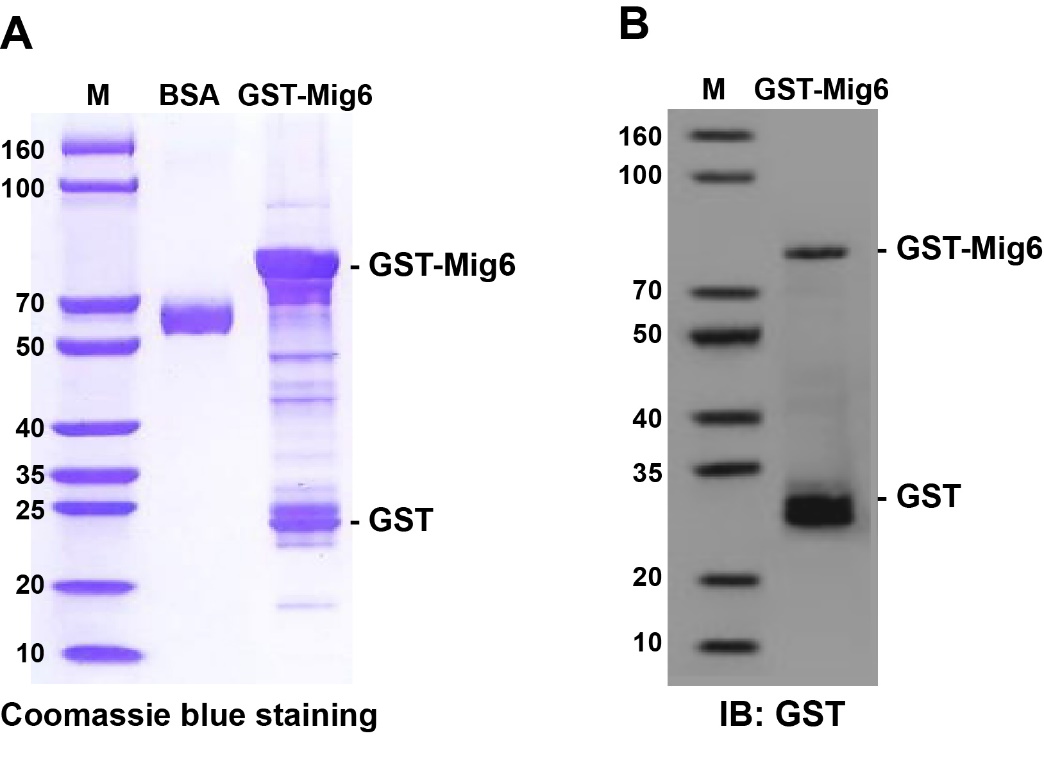
**

**Supplementary Figure 4.** GST-Mig6 fusion protein expression. (**A**, **B**) GST-fusion protein of Mig6 was expressed and purified from *E. coli*. GST-Mig6 fusion protein was validated by (**A**) Coomassie blue staining and (**B**) Western blot.


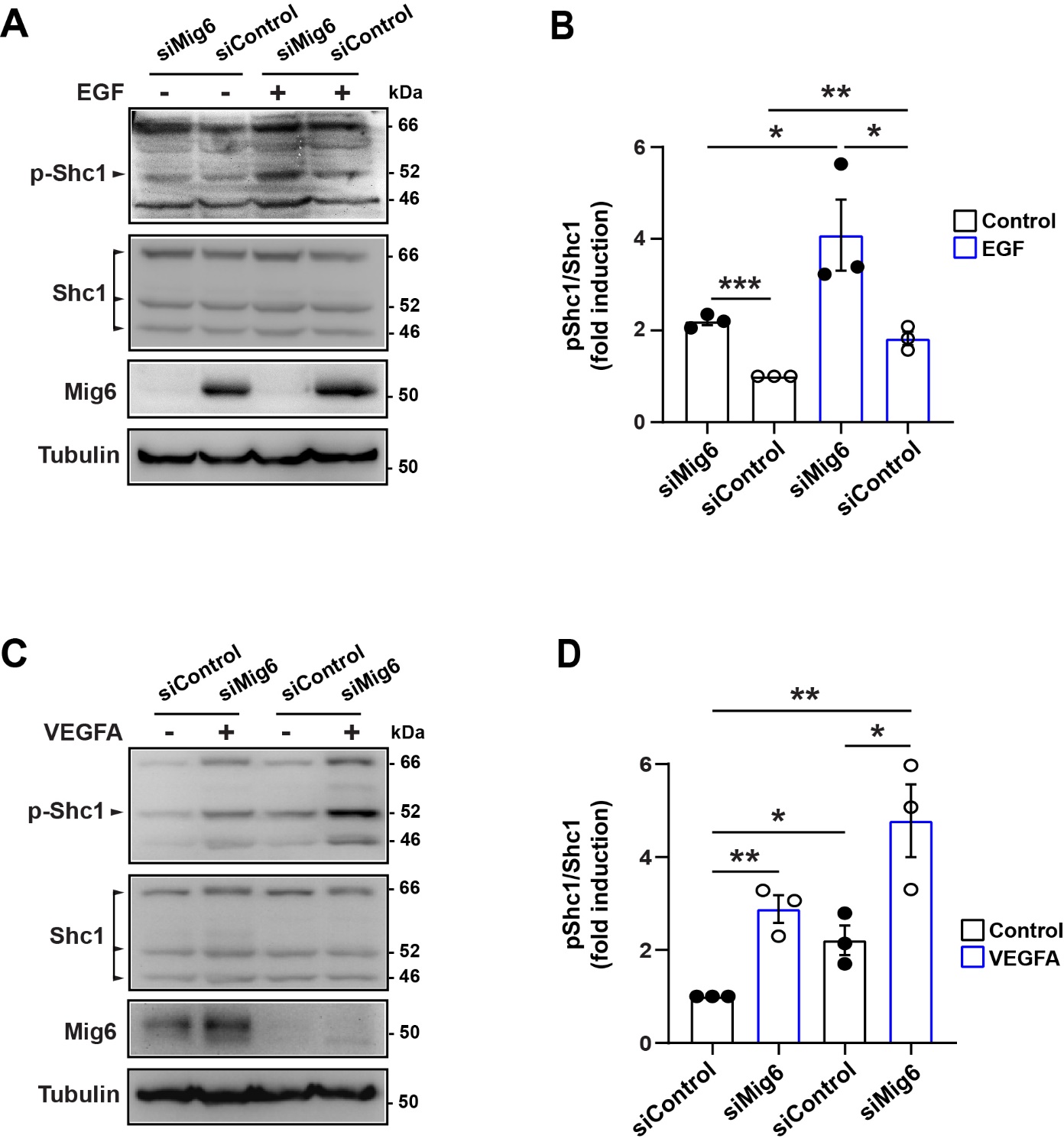


**Supplementary Figure 5.** Mig6 knockdown in HREC upregulates Shc1 phosphorylation. (**A, C**) Western blots showing that Mig6 knockdown increases Shc1 phosphorylation in HRECs both at the baseline level and in the presence of EGF (A) or VEGFA (C). (**B, D**) Tyrosine phosphorylation of Shc1 was analyzed by densitometry and normalized by total Shc1. Fold induction relative to the siControl is shown. The graph represents the mean ± SEM from three independent experiments. * *p* < 0.05, ** *p* < 0.01, *** *p* < 0.001 (paired Student’s t-test, two-tailed).
